# Diversifying the secretory routes in neurons

**DOI:** 10.3389/fnins.2015.00358

**Published:** 2015-10-07

**Authors:** José I. Valenzuela, Franck Perez

**Affiliations:** ^1^Cell Biology Department, Institut Curie, PSL Research University, UMR144Paris, France; ^2^Dynamics of Intracellular Organisation, Centre National de la Recherche Scientifique -UMR144Paris, France

**Keywords:** secretory route, endoplasmic reticulum, Golgi apparatus, Golgi outposts, protein trafficking, neuronal trafficking

## Abstract

Nervous system homeostasis and synaptic function need dedicated mechanisms to locally regulate the molecular composition of the neuronal plasma membrane and allow the development, maintenance and plastic modification of the neuronal morphology. The cytoskeleton and intracellular trafficking lies at the core of all these processes. In most mammalian cells, the Golgi apparatus (GA) is at the center of the biosynthetic pathway, located in the proximity of the microtubule-organizing center. In addition to this central localization, the somatic GA in neurons is complemented by satellite Golgi outposts (GOPs) in dendrites, which are essential for dendritic morphogenesis and are emerging like local stations of membranes trafficking to synapses. Largely, GOPs participation in post-ER trafficking has been determined by imaging the transport of the exogenous protein VSVG. Here we review the diversity of neuronal cargoes that traffic through GOPs and the assortment of different biosynthetic routes to synapses. We also analyze the recent advances in understanding the role of cytoskeleton and Golgi matrix proteins in the biogenesis of GOPs and how the diversity of secretory routes can be generated.

## Introduction

The directional relay of information in the nervous system requires morphologically asymmetric synaptic contacts. Neurons are highly polarized cells composed of two major functional domains: the somatodendritic compartment, responsible for receiving signals, and the axonal compartment, responsible of transmitting information. In addition, neurons develop a diversity of functional and morphologic subdomains (e.g., dendritic spines, synaptic buttons, Ranvier nodes, etc.), which implies that, throughout their lifetime, neurons need to precisely control their local molecular composition. Neurons mature from small symmetrical progenitors and increase by more than 200 folds their plasma membrane area to reach cellular surfaces more than 10,000 times bigger than a typical epithelial cell (Horton and Ehlers, 2004). The intracellular machinery involved in the acquisition, maintenance and plastic modification of neuronal morphology depends on the proper coordination of the cytoskeleton and the intracellular membrane trafficking.

During neuronal development, fundamental processes such as axonal specification and differential outgrowth of dendrites and axons critically depend on the supply of proteins and lipids through the secretory route. It is composed of well-organized compartments that include the endoplasmic reticulum (ER), the ER-to-Golgi intermediate compartment (ERGIC), the Golgi apparatus (GA) and the trans-Golgi network (TGN), in addition to membrane-bound intermediates that allow transport between compartments in a sequential and finely controlled way. In morphologically non-differentiated neurons, the selective supply of post-Golgi vesicles to one particular neurite precedes its specification as the future axon (Bradke and Dotti, 1997). Perturbing the secretory pathway modulates axonal growth. For example, inhibiting the activity of the Sar1 small GTPase, a primary component for the COPII-mediated vesicular export from ER, generates neurons with smaller axons while its overexpression produces neurons with longer axons (Aridor and Fish, 2009). After axon specification, inhibition of the secretory pathway by expressing a GTPase deficient mutant of the Arf1 small GTPase, or by blocking the post-Golgi trafficking using a kinase-dead mutant of protein kinase D1 (PKD1), results in a decrease of dendrites outgrowth (Horton et al., 2005). Interestingly, a genetic screen carried out in *Drosophila* identified the homologs of *Sec23, Sar1*, and *Rab1* as essentials for dendritic arbors outgrowth, but not for axons (Ye et al., 2007). These proteins are important for the ER-to-Golgi transport mediated by COPII vesicles, hence revealing a differential susceptibility of dendrites and axons to perturbations of membrane traffic during development. Inhibition of the secretory pathway in mature neurons still decreases the average total dendritic length, indicating it is required to maintain the dendritic arbor (Horton et al., 2005).

The number and density of neurotransmitter receptors control the potency of synapses. Neurotransmitter receptors supply thus needs to be regulated with a high spatiotemporal precision (Kennedy and Ehlers, 2006). Although endocytosis and recycling of synaptic receptors has been extensively studied, little is known about their site of synthesis and secretory transport. In this context, two neighbor synapses, separated by a few micrometers, may present a very different protein landscape at steady state. For example, one single spine contains between several tens to several hundreds of glutamate receptors. Thus, addition or removal of just a few receptors from the synaptic surface may be enough to elicit changes in the neurotransmission (Newpher and Ehlers, 2008) indicating that a tight control of secretion and endocytosis has to be established. Indeed, the long-term potentiation and the NMDA-induced increase of AMPA receptors (AMPARs) expressed at the plasma membrane directly depend on the secretory transport of AMPARs (Broutman and Baudry, 2001), highlighting the relevance of intracellular trafficking in neuronal physiology.

Here, we analyze the particular organization of the secretory pathway in neurons, the different possibilities of cargo trafficking that it offers and review recent evidences that help to understand how this diversity is generated.

## Organization of secretory routes in dendrites

In neurons, the general principles underlying the control of the secretory pathway applies, but the arrangement of secretory organelles presents unique particularities in terms of the enormous distances involved and the distribution of these organelles, particularly the GA (Horton and Ehlers, 2004; Ramírez and Couve, 2011).

As in any eukaryotic cell, the starting point of the secretory route is the ER, where the synthesis of most of membrane and secreted proteins occurs. Electron microscopy (EM) studies have reported the presence of a continuous endomembrane network of ER that spans the neuronal arborescence including soma, dendrites, axons, and in some cases reaching the inner of dendritic spines (Tsukita and Ishikawa, 1976; Broadwell and Cataldo, 1983; Spacek and Harris, 1997; Gardiol et al., 1999). Regions with a higher complexity of ER network have been described at dendritic branch points and near dendritic spines (Cui-Wang et al., 2012). The ER found in the soma is mainly composed of sheets of ribosome-decorated rough ER, while in dendrites the ER is constituted mostly by tubules of smooth ER running in parallel to the dendritic shaft with only few ribosomes attached (Broadwell and Cataldo, 1983; Martone et al., 1993; Krijnse-Locker et al., 1995; Spacek and Harris, 1997; Cooney et al., 2002). mRNAs translation of transmembrane proteins have been observed in dendrites and specialized compartments such as ERES, have been shown to be functional in the dendritic arbor (Gardiol et al., 1999; Aridor et al., 2004; Holt and Schuman, 2013).

The ERGIC is composed of long-lived structures that constitute sorting stations of anterograde and retrograde cargoes interconnected by highly mobile short-lived elements (Ben-Tekaya et al., 2005; Appenzeller-Herzog and Hauri, 2006). Several ERGIC markers are present in dendrites (Krijnse-Locker et al., 1995; Torre and Steward, 1996; Gardiol et al., 1999) forming stationary and mobile tubulo-vesicular structures whose distribution reaches territories distant from the soma (Hanus et al., 2014).

The GA is the main station of posttranslational modification, maturation and sorting. It consists of a polarized arrangement of stacked cisternae where occurs transport of proteins in lipids emanating from the ER and destined to post-Golgi station and the simultaneous retrograde flow of cargoes toward the ER (Farquhar and Palade, 1998; Johannes and Popoff, 2008). In neurons, as in many animal cells, the GA is found in a perinuclear area. In addition, a set of satellite Golgi outposts (GOPs) is present in around 18% of dendrites of matures neurons (Horton et al., 2005). 70–80% of mature hippocampal neurons display GOPs, which are preferentially localized to first-order segment of the apical dendrite in the cortex or to its corresponding major dendrite in hippocampal neurons in culture (Horton and Ehlers, 2003; Horton et al., 2005; Quassollo et al., 2015). They are discrete structures, discontinuous with the somatic Golgi, and some of them are composed of stacked cisternae (Horton and Ehlers, 2004; Pierce et al., 2001). Markers of *cis, medial*, and *trans* Golgi compartments have been detected in dendrites by immunofluorescence and EM (Pierce et al., 2001; Horton et al., 2005). Additionally, ER- and Golgi-dependent protein glycosylation can be carried out in isolated dendrites (Torre and Steward, 1996). Recent evidences point to the existence of punctate single-compartment GOPs (scGOPs) in dendritic shafts of *Drosophila* neurons *in vivo* in which *cis, medial, and trans* cisternae are thought to be disconnected from each other. This contrasts to multi-compartment GOPs (mcGOPs), localized in both shafts and dendritic branch points, which are organized as stacks in a GM130-dependent manner (Zhou et al., 2014). Further research will be needed to decipher whether the proposed scGOPs are transport carriers cycling within the secretory pathway or effectively correspond to *bona fide* GOPs.

**Figure 1:**
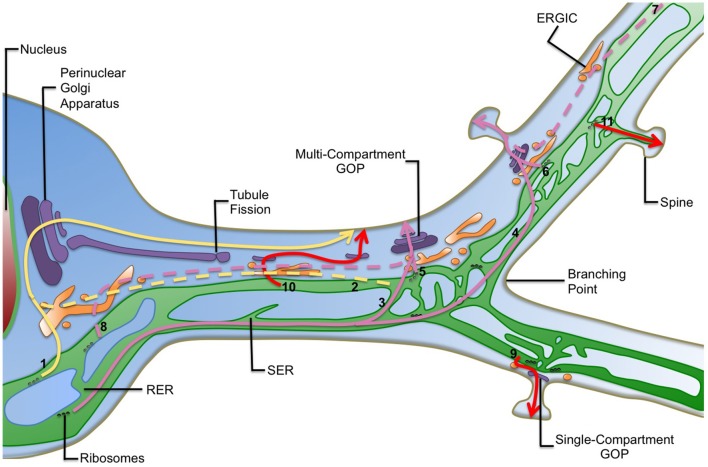
**Diversity of the secretory routes in dendrites**. Known and suspected routes to the plasma membrane in dendrites are shown. For simplicity post-Golgi carriers are not shown. In the canonical secretory route (yellow arrows) cargoes can be synthetized and exported from the somatic endoplasmic reticulum (ER) and transported through the ER-to-Golgi intermediate compartment (ERGIC) in the cell body to the perinuclear Golgi apparatus (1). Alternatively they can be exported distally in the dendritic ER and use long-haul ER-to-Golgi transport (2, dashed lines) to reach the somatic Golgi apparatus. Several possibilities of local secretory trafficking coexist in neurons (pink arrows): protein cargoes can be synthetized within the rough endoplasmic reticulum (RER) in somas and be transported over long-distances through the dendritic smooth endoplasmic reticulum (SER). They can then be preferentially exported from the ER at dendritic branching points (3) or synapses (4) where local zones of ER complexity confine cargo mobility and would favor secretion through GOPs. Cargo proteins can be also synthetized in ER-associated ribosomes in dendrites and transported locally using satellite GOPs. Export to GOPs will occur mainly at dendritic branching points (5) and synaptic contacts (6) where they are enriched and seems to support the dynamics of dendritic arborization and the synaptic delivery of neurotransmitter receptors respectively. Additionally, cargoes can use long-haul ER-to-Golgi transport before reaching GOPs (pink dashed lines). Subsequently, synaptic activity may restrict the scale of post-ER trafficking. Therefore, cargoes can be exported from the dendritic ER far away from GOPs (7) or from the somatic ER and travel in post-ER carriers to GOPs (8). Some more hypothetical secretory routes are also depicted (red arrows): the presence of single-compartment GOPs raises the possibility that particular cargoes may use only one Golgi compartment (*cis, medial*, or *trans*) in their way to the plasma membrane (9). Alternatively, they may sequentially use separated Golgi compartments before reaching their final destination (10). These options may increase the diversity of posttranslational modifications patterns of cargoes. Additionally, the ER dynamically explores the neck of dendritic spines, suggesting that cargoes may be transferred directly or indirectly from the ER to synapses within spines (11). Indeed, a specialization of the ER, called spine apparatus, has been reported in spines. Finally, GOPs formation may be driven by fission from the perinuclear Golgi, which would then be transported to remote areas, allowing the transport of large cargo loads within fissioned Golgi stacks to distal dendrites.

The overall organization of secretory organelles in neurons allows the co-existence of two major routes for the secretory trafficking of proteins and membranes in dendrites, namely canonical and local routes that differ in the alternative usage of somatic GA or GOPs, respectively (Figure 1; Horton and Ehlers, 2003; Ramírez and Couve, 2011). The canonical trafficking is similar to the trafficking occurring in non-neuronal cells: newly synthesized secretory proteins are transported from the ER to the perinuclear GA where they mature to be sorted in post-Golgi intermediates to their insertion/secretion sites in later compartments (the plasma membrane, endosomes, lysosomes, etc.). In the local route, nascent secretory proteins are locally translated into dendritic ER (dER) structures, or transported inside the dER to peripheral sites. There, they are transported from the dER to satellite GOPs, hence establishing a local trafficking route for these cargoes in the dendritic compartment.

## Diversity of secretory routes and cargoes in neurons

ER-to-Golgi transport has been classically studied using the thermosensitive mutant of VSVG (VSVG^ts045^), a viral glycoprotein which is retained within the ER at the restrictive temperature of 39.5°C. Switching to the permissive temperature of 32°C enables its rapid export from the ER and synchronous transport to the cell surface (Presley et al., 1997; Scales et al., 1997). In neurons, live-cell imaging have shown that GFP-tagged VSVG^ts045^ is exported from the dER and a fraction of post-ER carriers are fused with GOPs (Horton and Ehlers, 2003), suggesting that protein processing and secretion can be carried out far from the soma. Importantly, blocking Golgi export at 20°C accumulates VSVG^ts045^ in dendritic branching points in primary neurons (Horton et al., 2005; Cui-Wang et al., 2012). Mannosidase II-positive GOPs are enriched in dendritic branching points of *Drosophila* neurons *in vivo* and their photo-ablation inhibits the dynamics of extension and retraction of dendritic branches. This suggests that GOPs locally support the membrane turnover required for dendritic remodeling (Ye et al., 2007), although this may also be explained by an indirect effect on microtubules polymerization in dendritic branching points (Ori-McKenney et al., 2012; Nguyen et al., 2014; Zhou et al., 2014). Nevertheless, in mammalian neurons, Golgi-dependent trafficking supports dendrites outgrowth and the complete biosynthetic machinery is enriched in dendritic branch points (Cui-Wang et al., 2012; Horton et al., 2005). Indeed, ER and Golgi export of VSVG^ts045^ occurs preferentially in dendritic branching points (Cui-Wang et al., 2012; Horton et al., 2005) and local ER-release of a light-controlled VSVG leads to its accumulation within branch points, presumably in GOPs (Chen et al., 2013). Moreover, dendritic branching points represent hot spots of exocytosis for VAMP2 (Cui-Wang et al., 2012).

VSVG^ts045^ has been essential to unravel dendritic secretory routes, but it is an exogenous protein that represents only one category of cargo, limiting further physiological interpretations. Diverse cargo proteins needs dedicated routes to specifically manage their transport (Boncompain and Perez, 2013). For example, in contrast to VSVG^ts045^, the voltage-gated potassium channel Kv4.2, which is crucial for the repolarization phase of action potentials, binds to its auxiliary subunit KChIP1 and traffic in COPI-dependent and COPII-independent post-ER carriers (Hasdemir et al., 2005). In hippocampal neurons, KChIP1 accumulates in GOPs (Hasdemir et al., 2005), highlighting the relevance of studying not only model cargos but also neuronal relevant proteins. Another neuronal cargo is the brain-derived neurotrophic factor (BDNF), which has major roles in neuron survival, differentiation, dendritic outgrowth, synaptic formation, and memory. At 20°C BDNF localizes in both the somatic Golgi and GOPs (Horton and Ehlers, 2003) suggesting that remote BDNF secretion could locally regulate synaptic remodeling in nearby dendritic territories.

Biosynthesis at- and transport through- the dER is closely linked to local trafficking, constituting an early stage of protein sorting and synaptic regulation of membrane trafficking (Aridor et al., 2004; Jeyifous et al., 2009; Ramírez and Couve, 2011; Cui-Wang et al., 2012; Valenzuela et al., 2014). An example is the subunit α7 of nicotinic acetylcholine receptor, the trafficking of which can be locally coordinated in dendrites ER: at high levels, the Ric3 chaperone promotes ER retention and transport of α7-receptors within the dER, while at low levels it stimulates the assembly, ER release and surface expression of α7-receptors (Alexander et al., 2010). Another example is the GABA_B_ receptor (GABA_B_R), an heterodimeric G protein-coupled receptor (GPCRs) that regulates the slow and tonic inhibition in the brain. The GABA_B1_ subunit has an ER retention signal (Margeta-Mitrovic et al., 2000), which allows its long-range transport through the dER by a mechanism that, in addition to diffusion, involves microtubule-dependent transport (Ramírez et al., 2009; Valenzuela et al., 2014). The GABA_B_R traffics through both somatic and dendritic Golgi to the somatodendritic membrane (Valenzuela et al., 2014). Visualization of synchronized ER-to-Golgi trafficking of a GABA_B1_ mutant that lacks of the ER retention signals, revealed carriers traveling over a long distance prior to fusion with GOPs, suggesting that local dER export does not necessarily determine a local trafficking through GOPs and that different spatial ranges of ERGIC transport can regulate the local protein supply (Hanus et al., 2014; Valenzuela et al., 2014).

It has been proposed that bidirectional ER-to-Golgi transport constitutes a checkpoint to modulate the balance between local vs. long-range trafficking in dendrites (Hanus et al., 2014). The serotonin receptor 5-HT_1A_R, which is a main target of psychotropic and antidepressant molecules, is transported to dendrites in a complex with Yip1A, Rab6 and molecular motors Kif5B and dynein (Carrel et al., 2008; Al Awabdh et al., 2012) suggesting that ER-to-GOPs trafficking is necessary for the transport of 5-HT_1A_R to distal dendrites. It interacts with Yif1B that regulates specifically its anterograde ER-to-Golgi transport (Alterio et al., 2015). Interestingly, the amyotrophic lateral sclerosis related protein VAPB interacts with Yif1A. Although knockdown of either VAPB or Yif1A does not affects 5-HT_1A_R transport, both proteins are required for the trafficking to dendrites of the transmembrane protein GFP-CD8 and for normal dendrite morphology (Kuijpers et al., 2013).

A fundamental issue is whether canonical and local routes present selectivity for a particular subset of cargoes. For example, AMPARs seems to be sorted apart from NMDA receptors (NMDARs) within the somatic ER. NMDARs are then transported by the kinesin Kif17 in a dER subcompartment as a complex with scaffold proteins CASK and SAP97, which leads to the exclusive fusion of pre-Golgi NMDARs carriers with GOPs (Jeyifous et al., 2009). Consistent with this local route, NMDARs accumulate in dendritic ERES far away from the soma after group I metabotropic glutamate receptors (mGluRs) stimulation (Aridor et al., 2004). Activity-dependent control of mRNA splicing or interaction with the chaperone BIP (for NR1 or GluN2A subunits respectively) regulates the supply of NMDARs from the dER to synapses, which is relevant for memory formation (Mu et al., 2003; Zhang et al., 2015). In contrast, AMPARs seem to use specifically the canonical secretory route (Jeyifous et al., 2009). This specific sorting seems to depend on the conformation of the MAGUK-family protein SAP97, which associates with AMPARs early in the secretory pathway (Sans et al., 2001). Upon CASK binding, the conformation of SAP97 is altered, allowing its association to NMDARs and their sorting to GOPs (Jeyifous et al., 2009; Lin et al., 2013). ADAM10, the enzyme responsible for the α-secretase cleavage that prevents the formation of β–amyloid in neurons, associates with SAP97 in a protein kinase C (PKC) dependent fashion, which is essential for ADAM10 trafficking from GOPs to synapses, but not from the ER to GOPs (Saraceno et al., 2014). Importantly, regulatory proteins seem to be involved in the local transport of several cargos. For example, Kif17 participates to the transport of NMDARs, Kv4.2 and VSVG^ts045^ (Setou et al., 2000; Chu et al., 2006; Hanus et al., 2014), raising the question of whether Kif17 is a main microtubule-dependent motor for pre-Golgi carriers during local trafficking. It will be interesting to examine if other Kif17 cargoes such as kainate receptors, also use local trafficking (Kayadjanian et al., 2007).

An outstanding question is whether the usage of GOPs is a wide mechanism for dendritic membrane transport. Notably, BDNF, VSVG^ts045^, and GABA_B_Rs have been shown to be able to use both canonical and local trafficking paths (Horton and Ehlers, 2003; Valenzuela et al., 2014). An attractive possibility is that synaptic activity modulates the alternative usage of these pathways. In this context, mGluRs signaling locally confines the cargo mobility within the dER, increasing secretion by a mechanism involving PKC and the ER protein CLIMP63 (Cui-Wang et al., 2012). Similarly, ionotropic glutamate receptor activation restricts the spatial range of post-ER carriers transport in a CaMKII and Kif17 dependent fashion. This increases VSVG^ts045^ delivery to the plasma membrane, with a minor effect on cargo diffusion within the dER (Cui-Wang et al., 2012; Hanus et al., 2014), revealing a functional link between synaptic activity and the early secretory pathway in dendrites.

## Generating the trafficking diversity: GOPs biogenesis

GOPs formation is developmentally regulated in hippocampal neurons, meaning that it steadily increases during neuronal growth and differentiation (Horton and Ehlers, 2003; Horton et al., 2005). Two main options could explain the biogenesis of GOPs: (i) a local GOPs production from the dER or (ii) a somatic Golgi fragmentation and subsequent dispersion into dendrites. Recently, it has been shown that GOPs destined to the major dendrite are generated by a sequential process that involves polarized deployment and fission of tubules derived from the somatic GA, which are then transported and condensed in dendrites (Quassollo et al., 2015). In *Drosophila* neurons GOPs are transported by dynein through their interaction with the golgin Lava lamp (Lva; Ye et al., 2007; Zheng et al., 2008). Parkinson's disease-associated kinase Lrrk binds to- and phosphorylates Lva, inhibiting GOPs movement due to Lva/dynein dissociation (Lin et al., 2015). GOPs formation is controlled by the RhoA-Rock pathway through key components of the Golgi fission machinery, including the kinases LIMK1 and PKD1 which regulate the activity of the actin dynamizing factor Cofilin, its activating phosphatase slingshot-1, and dynamin-2 (Quassollo et al., 2015). Activation or inactivation of this pathway within the GA regulates GOPs biogenesis.

In *Drosophila* neurons, GM130 is both necessary and sufficient to generate mcGOPs (Zhou et al., 2014). Interestingly, scGOPs move faster than mcGOPs raising the question of whether GM130 is needed for GOPs transport or for condensation in dendrites (Zhou et al., 2014). During GOPs generation, some Golgi-emanating tubules contain markers of proximal and distal cisternae, implying that mcGOPs could be directly derived from the somatic GA (Quassollo et al., 2015). Interestingly, the RhoA-Rock pathway is not involved in Golgi-derived tubule formation, elongation or polarized distribution, suggesting that additional factors regulate these processes (Quassollo et al., 2015). Reelin stimulates Cdc42- or α-pix- mediated GA translocation into the apical dendrite (Matsuki et al., 2010; Meseke et al., 2013). This seems to be essential for apical dendrite orientation and dendritic development, suggesting that the reelin pathway may be involved in polarized Golgi-derived tubule deployment and GOPs generation during development (Meseke et al., 2013; Quassollo et al., 2015). Currently, the mechanisms of GOPs biogenesis in minor dendrites remain unknown. In this context, the overexpression of the Golgi structural protein GRASP65 causes Golgi dispersal into multiple dendrites, without inhibiting secretory trafficking, generating a marked reduction in the polarity of the dendritic arbor (Horton et al., 2005). Thus, Golgi stacking and ribbon linking may modulate GOPs formation in minor dendrites.

## Projections

The multiplicity of GOPs compositions confers a range of possibilities for routing different cargoes, contributing to diversify their output in terms of dynamics, localization, and post-translational modifications patterns. How to balance the usage of canonical and local routes and what determines the specific use of local routes, or the trafficking through a scGOP, are outstanding questions. The emerging possibility of *in situ* detection of *de novo* synthesis of any interest protein (tom Dieck et al., 2015), in combination with systems that allow trafficking synchronization of specific cargoes (Rivera et al., 2000; Boncompain et al., 2012; Chen et al., 2013) coupled to super-resolution microscopy (Maglione and Sigrist, 2013) will give us an unprecedented opportunity to decipher the diversity of secretory routes in highly differentiated structures like dendrites and axons. A fundamental issue is how trafficking routes contribute to the synaptic function and neuronal physiology, and conversely, how synapses control these secretory routes. Combination of trafficking synchronization assays with optogenetic tools for the local manipulation of synaptic activity arise as suitable approaches to gain a better understanding of how changes in intracellular transport events could lead to changes in cognition, emotions, memory, and learning.

## Funding

Institut Curie, CNRS, the French Agence Nationale de la Recherche (ANR-12-BSV2-0003-01), Fondation Recherche Médicale (DEQ20120323723), LabEx CelTisPhyBio (ANR-10-LBX-0038 part of the IDEX PSL no. ANR-10-IDEX-0001-02; to FP). Long-Term EMBO Fellowship (ALTF 607-2015) co-funded by the European Commission FP7 (Marie Curie Actions, LTFCOFUND2013, GA-2013-609409; to JIV).

### Conflict of interest statement

The authors declare that the research was conducted in the absence of any commercial or financial relationships that could be construed as a potential conflict of interest.
